# Malignant acrospiroma: a case report in the era of next generation sequencing

**DOI:** 10.1186/s12885-017-3217-5

**Published:** 2017-03-27

**Authors:** Maria Diab, Ali Gabali, Muaiad Kittaneh

**Affiliations:** 1 Department of Internal Medicine, Wayne State University, School of Medicine, Detroit, MI USA; 2 Department of Pathology, Wayne State University, School of Medicine, Detroit, MI USA; 3 Cardinal Bernardin Cancer Center, Loyola University Chicago Stritch School of Medicine, Maywood, IL USA

**Keywords:** Acrospiroma, Malignant, Acral, Next generation, Case report

## Abstract

**Background:**

Malignant acrospiroma is a rare tumor of the eccrine sweat glands accounting for around 6% of all malignant eccrine tumors. Typically, it presents as large ulcerated nodules, and diagnosis can be challenging as it has great overlap with its benign counterpart.

**Case presentation:**

We herein report a case of acral malignant acrospiroma, initially treated with surgical excision and adjuvant radiotherapy. After metastatic disease was confirmed, subject received multiple lines of chemo- as well as targeted therapy. Genomic testing was also done using next generation sequencing.

**Conclusion:**

To the best of our knowledge, this is the first case of acral malignant acrospiroma with reported next generation sequencing results.

## Background

Malignant acrospiromas arise from the intradermal duct of eccrine sweat glands and account for approximately 6% of malignant eccrine tumors [1] compared to their more prevalent benign counterparts [2]. They appear in the literature under multiple nomenclatures, including clear cell hidradenocarcinoma, malignant clear cell hidradenoma, and malignant hidradenoma [3, 4]. Disease usually manifests in middle-age [5]. Some reports show disease is more common in females [6], but there does not seem to be an obvious gender predominance [4, 7, 8]. Lesions typically present as slow growing nodules that can ulcerate and drain [6, 9]. They range in size from 0.5 to 10 cm [6, 9]. They usually involve the head, neck, and extremities; less common sites include the chest and breasts [1, 4, 5, 9, 10].

This tumor has an aggressive behavior with more than 50% local recurrence rates [11]. Wide surgical excision is the treatment of choice [12–14]. The efficacy of adjuvant chemotherapy is controversial. Adjuvant radiotherapy has been shown successful in some cases [4, 12, 13]. With the advent of targeted therapy, genetic profiling is becoming a more attractive tool. EGFR overexpression was observed by Piris et al. in 3 out of 12 malignant hidradenomas [15]. However, there has been no reports of next generation sequencing. We herein report a case of acral malignant acrospiroma initially treated with surgical excision and adjuvant radiotherapy. After metastases was discovered, subject received multiple lines of chemotherapy. We also tested the tumor for genomic alterations using next generation sequencing.

## Case presentation

**Fig. 1:**
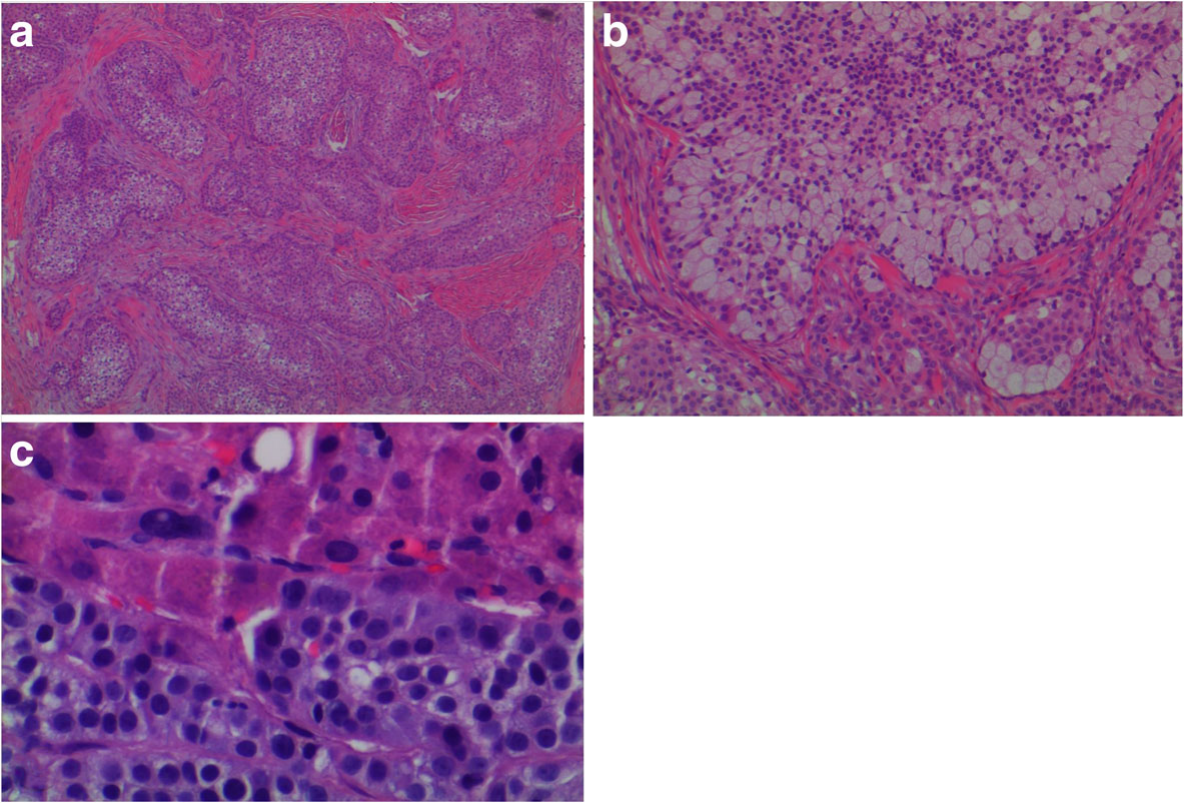
**a** Malignant cells are arranged in cords and sheets separated by a markedly desmoplastic stroma and extending deep into the dermis (H&E at 40X magnification). **b** Neoplastic cells showing squamous, sebaceous and mucinous differentiation. (H&E at 100X magnificent). **c** Liver tissue (upper) with metastatic malignant acrospiroma (lower). The neoplastic cells have uniformly hyperchromatic nuclei (H&E at 400X magnification)

A 73-year-old White male was referred to our institution for the treatment of metastatic malignant acrospiroma. He initially presented in 2009 with a nodular lesion on the dorsal aspect of the left great toe. An excisional biopsy of the lesion showed subcutaneous tissue containing multilobular and ill-defined tumor. The pathologic diagnosis was consistent with acrospiroma; margins were involved (Fig. 1a and b). Wide surgical excision was subsequently performed to clear the margins and the patient was followed by clinical surveillance. Six months later, the patient developed local recurrence and the pathological findings were similar to the original biopsy. At this time, he was treated with radiation therapy (received 40 Gray) followed by clinical surveillance again.

Three years later, he presented with left groin mass. A positron emission tomography-computed tomography (PET-CT) scan showed uptake in the left inguinal lymph nodes and multiple hepatic lesions. A biopsy from the hepatic lesion was consistent with metastatic acrospiroma (Fig. 1c). His laboratory studies were within normal limits, except for an elevated carcinoembryonic antigen (CEA) of 206.1 ng/mL (normal is less than 5.1 ng/mL). Patient was subsequently started on systemic chemotherapy. He was initially treated with capecitabine. After 2 months of therapy, however, disease progressed and treatment was discontinued. The second line of chemotherapy utilized carboplatin. He completed a total of 6 cycles on this combination until disease progression. On progression, he was referred to our institution for consideration of experimental therapy. Tissue from hepatic metastasis was sent for genomic profiling using next generation sequencing Knight diagnostic gene panel. Unfortunately, no actionable genomic alterations were identified. Genes tested included EGFR, ALK, BRAF, JAK2, NOTCH1, NRAS, or PTEN (Table 1), and the sequencing coverage ranged from 92.4 to 100%. Our patient was then placed on a phase I trial utilizing a PI3 kinase AkT mTOR inhibitor and ultimately progressed and succumbed to his disease.

**Table 1:** Mutation screening by next generation sequencing on tissue from hepatic metastasis∞

Gene	Percentage of expected sequencing coverage*
AKT1, BRAF, CDKN2A, DDR2, EGFR, ERBB2, HRAS, JAK2, KDR, KRAS, MAP2K1, NRAS, NTRK1, NTRK2, PIK3R1, PTPRD, TP53	100%
PIK3CA	96.9%
NOTCH1, NTRK3	96%
ALK	95%
PTEN	92.4%
PIK3R2	78.8%

*∞ No mutations were identified*

*Percent of gene covered by a minimum of 100 sequence reads, as compared with expected coverage based on data from 10 normal DNA samples

## Pathology

Sections from the initial diagnostic biopsy revealed tumor that composed of epithelial cells with foci of clear cell morphology and cystic changes, suggesting trichilemmal differentiation (Fig. 1a and b). Some foci exhibited a sclerotic stroma with cystic changes and others showed mucinous stroma and mucinous changes within the epithelial cells (mucinous metaplasia). There was no ductal differentiation. The tumor showed pushing boarders, focal infiltrative pattern and small areas of increased pleomorphic features and atypia with mitotic activity and focal necrosis. The surrounding connective tissue margins were involved. No immunohistochemical studies were performed and the pathological diagnosis was that of acrospiroma with atypia suggesting foci of malignant transformation. Apart from metastasis to liver, all subsequent metastasis sites, focal and distant, showed findings similar to those seen in the original biopsy. In a specimen that showed liver metastasis (Fig. 1c), the tumor exhibited more cells with clear cytoplasm and hyperchromatic nuclei. Interspersed with the clear cells is a small population of goblet cells which appear to contain mucinous materials. Because of the clear cell morphology in a small population, CD10 immunohistochemical stain was also performed to exclude clear cell type renal cell carcinoma, and it was negative.

## Discussion and conclusion

Malignant acrospiroma is a rare tumor of the eccrine sweat glands that usually displays an aggressive behavior. Disease can arise de novo or transform from a previous benign lesion. Our patient did not have a history of prior malignancy. One of the challenges in establishing the disease is that it is often mistaken for benign lesions, and patients are monitored for some time before tumors cause symptoms that prompt seeking medical attention. Wenzel described a case of malignant acrospiroma that was initially mistaken for osteomyelitis. The patient was initially started on antibiotics before pathology on the surgically excised digit confirmed malignancy [14]. In other instances, disease masquerades as more common malignant lesions, like malignant melanoma [16]. The other challenge lies in complete sampling of the lesion. Disease might be focal and/or may reside in the periphery of the biopsied sample.

Wide surgical excision is the mainstay of treatment [12]. Wildemore compared outcomes with Mohs micrographic surgery to conventional surgery; all 19 patients who underwent Mohs micrographic surgery had no local recurrence at a 29-month follow up [16]. Our patient was initially treated with surgical excision after the initial biopsy showed positive margins. Even wide surgical excision, recurrence rates of as high as 50% have been documented [17]. Due to the aggressive nature and the tendency for lymphatic invasion, some authors advocate for prophylactic lymph node dissection [18]. Evidence is lacking on the efficacy of adjuvant chemotherapy [19, 20]. Furthermore, whether the reported results are due to a real response to chemotherapy or due to a slow growth of the tumor is questionable. In a case of metastatic disease, complete response was achieved after 3 months of capecitabine (1500 mg/m^2^ in a split daily dose, on a 3-weeks-on/1-week-off schedule) [21]. Treatment was complicated with grade 2 fatigue. At 24-month follow up, Lerner’s patient was disease free. The use of second-line sunitinib was associated with an 8-month progression-free survival in one patient [22]. The role of radiotherapy appears to be more established [4, 12, 13]. In one report of 3 patients who received adjuvant radiotherapy, 2 patients remained disease free at 27 and 35 months, respectively, after the completion of treatment; the third died of rapidly progressive systemic disease [23]. The dose of radiation was 70 Gray for the surgical bed and 50 Gray for the regional lymphatic chains.

With the advent of targeted therapy, the use of genomics in the management of tumors has gained popularity. Several mutations have been previously identified in different cutaneous neoplasms, including t(11;19) in clear cell hidradenomas, hidradenocarcinomas, and mucoepidermoid carcinomas [2, 24, 25], resulting in a TORC1-MAML2 gene fusion; TP53 mutations and amplification of the Her2/neu gene in hidradenocarcinomas [25]; and BRAF-V600E in aggressive digital papillary adenocarcinoma [26]. Despite reports of using next generation sequencing in other cutaneous neoplasms, there have been no reports on its application in malignant acrospiroma. Unfortunately, although next generation sequencing was conducted in our case, it failed to show mutations that would mandate targeted therapy. Furthermore, none of the aberrations described in other cutaneous tumors were identified.

Our patient was treated with chemotherapy when his disease metastasized. He then entered a phase I trial utilizing a PI3 kinase AkT mTOR inhibitor. The patient was treated on a phase I PI3K/AKt clinical trial that didn’t require PI3K/AKt dysregulation. This was a dose finding study and allowed patients with any tumor type regardless of their PI3K/AKt mutation status to be included. The choice of PI3K/Akt was arbitrary as this is a commonly dysregulated pathway in cancer, and at the current time there is no single test available to predict PI3K/AKt dysregulation or response to Pi3K/Akt inhbitors. However, he ultimately progressed and succumbed to his disease. The lack of identified mutations might explain the aggressive nature of the disease.

Malignant acrospiroma is a rare tumor that originates from the eccrine sweat glands. Aggressive in its nature, its diagnosis is challenging, and specific tumor markers and gene mutations are not defined. Wide surgical excision, with or without prophylactic lymph node dissection, is the treatment of choice. Evidence is lacking on the efficacy and/or advised regimen of chemoradiotherapy. Prognosis is poor with systemic disease. More studies are needed for the management of this disease.

## Abbreviations

CEA: Carcinoembryonic antigen
Mg/m^**2**^: Milligrams per squared meters
Ng/mL: Nanograms per milliliter
PET-CT: Positron emission tomography-computed tomography

## References

[1] Mehregan AH, Hashimoto K, Rahbari H (1983). Eccrine adenocarcinoma. A clinicopathologic study of 35 cases. Arch Dermatol.

[2] Tingaud C (2016). Lymph node location of a clear cell hidradenoma: report of a patient and review of literature. J Cutan Pathol.

[3] Crowson AN, Magro CM, Mihm MC (2006). Malignant adnexal neoplasms. Mod Pathol.

[4] Long WP, Dupin C, Levine EA (1998). Recurrent malignant acrospiroma. Treatment by chest wall excision. Dermatol Surg.

[5] Gortler I (2001). Metastatic malignant acrospiroma of the hand. Eur J Surg Oncol.

[6] Kauderer C, Clarke HD, Fatone CT (1995). Malignant eccrine acrospiroma. A case study. J Am Podiatr Med Assoc.

[7] Cruz DJ (1987). Sweat gland carcinomas: a comprehensive review. Semin Diagn Pathol.

[8] Deckelbaum S (2014). Eccrine poromatosis: case report and review of the literature. Int J Dermatol.

[9] Ogilvie JW (1982). Malignant eccrine acrospiroma. A case report. J Bone Joint Surg Am.

[10] Cyrlak D, Barr RJ, Wile AG (1995). Malignant eccrine acrospiroma of the breast. Int J Dermatol.

[11] Wilson KM, Jubert AV, Joseph JI (1989). Sweat gland carcinoma of the hand (malignant acrospiroma). J Hand Surg [Am].

[12] Andreoli MT, Itani KM (2011). Malignant eccrine spiradenoma: a meta-analysis of reported cases. Am J Surg.

[13] Bandyopadhyay A (2013). Malignant acrospiroma of chest and abdominal wall treated with chemotherapy. Indian J Dermatol.

[14] Wenzel E (2012). Malignant eccrine acrospiroma: a case report. J Am Podiatr Med Assoc.

[15] Piris A (2010). Epidermal growth factor receptor gene status by fluorescence in situ hybridization in malignant, atypical, and benign hidradenomas. Am J Dermatopathol.

[16] Wildemore JK, Lee JB, Humphreys TR (2004). Mohs surgery for malignant eccrine neoplasms. Dermatol Surg.

[17] Keasbey LE, Hadley GG (1954). Clearcell hidradenoma; report of three cases with widespread metastases. Cancer.

[18] El-Domeiri AA (1971). *Sweat gland carcinoma: a clinico-pathologic study of 83 patients*. Ann Surg.

[19] Kersting DW (1963). Clear cell hidradenoma and hidradenocarcinoma. Arch Dermatol.

[20] Lopez-Burbano LF (1987). Malignant clear-cell hidradenoma. Plast Reconstr Surg.

[21] Lerner A (2011). Complete response of metastatic malignant hidradenocarcinoma to capecitabine treatment. Arch Dermatol.

[22] Battistella M (2010). Sunitinib efficacy in the treatment of metastatic skin adnexal carcinomas: report of two patients with hidradenocarcinoma and trichoblastic carcinoma. J Eur Acad Dermatol Venereol.

[23] Harari PM (1990). The role of radiotherapy in the treatment of malignant sweat gland neoplasms. Cancer.

[24] Behboudi A (2005). Clear cell hidradenoma of the skin-a third tumor type with a t(11;19)--associated TORC1-MAML2 gene fusion. Genes Chromosom Cancer.

[25] Kazakov DV (2009). Cutaneous hidradenocarcinoma: a clinicopathological, immunohistochemical, and molecular biologic study of 14 cases, including Her2/neu gene expression/amplification, TP53 gene mutation analysis, and t(11;19) translocation. Am J Dermatopathol.

[26] Bell D (2015). Next-generation sequencing reveals rare genomic alterations in aggressive digital papillary adenocarcinoma. Ann Diagn Pathol.

